# Best of both worlds? Helpers in a cooperative fairy-wren assist most to breeding pairs that comprise a potential mate and a relative

**DOI:** 10.1098/rsos.231342

**Published:** 2023-11-15

**Authors:** Niki Teunissen, Marie Fan, Michael J. Roast, Nataly Hidalgo Aranzamendi, Sjouke A. Kingma, Anne Peters

**Affiliations:** ^1^ School of Biological Sciences, Monash University, Clayton, Victoria, Australia; ^2^ Behavioural Ecology Group, Department of Animal Sciences, Wageningen University and Research, Wageningen, The Netherlands

**Keywords:** alloparental care, nestling provisioning, cooperation, helping, social environment, group composition

## Abstract

In cooperative breeders, individuals forego independent reproduction and help others raise offspring. Helping is proposed to be driven by indirect benefits from raising relatives, and/or direct benefits from raising additional recruits or helping itself. We propose that consideration of social context is also important, in particular the characteristics of the breeding pair: helping may also serve to lighten the workload of—or maintain social bonds with—breeders (e.g. kin, potential mates) who in turn can offer benefits to helpers (e.g. prolonged nepotism, future mating, future production of relatives). Here, we test this hypothesis, while controlling for potential direct and indirect benefits from raising offspring, in purple-crowned fairy-wrens (*Malurus coronatus*) exhibiting variation in social group composition, and thus, breeder value. We show that helper provisioning rates to the nest were explained by characteristics of breeders that helpers assisted, rather than benefits from raising offspring. The presence of at least one related breeder was a prerequisite to help, but helpers provisioned most if assisting a relative *and* potential mate. Neglecting to take group composition into account would have led to misinterpretation of our results. A comprehensive understanding of the evolution of cooperative breeding hence requires nuanced consideration of social context.

## 1. Introduction

Cooperative breeding, where subordinate individuals forego their own reproduction and instead help raise the offspring of others, is an important model system to study the evolution of costly, seemingly altruistic behaviour [1,2]. Kin selection has traditionally been considered a key explanation for the evolution of helping behaviour, since helpers can gain indirect benefits from increasing the production of related offspring [2–4]. While indirect benefits can clearly be important [5], the importance of direct benefits to helpers is increasingly recognized [6–8]. Direct benefits such as securing parentage as a subordinate, or future opportunities for reproduction via territory inheritance, have been shown to drive helping behaviour along with or instead of indirect benefits [9–11]. In fact, direct benefits can explain a substantial amount of variation in helping effort between cooperatively breeding birds [12]. This body of research has focused primarily on how helping may increase focal helpers' fitness via the production of additional recruits, or via immediate or future benefits directly to helpers (e.g. access to reproduction) [2,13,14].

Additionally, help benefits breeders: when breeders receive assistance with reproduction, their reproductive output is often increased [15–17] and their parental effort lowered (load-lightening; e.g. [18–21]), which can result in their improved survival and/or condition [16,20–22]. As a consequence, helpers may benefit from helping specific breeders, yet these benefits are not factored in broadly, beyond benefits deriving from indirect kin selection, when aiming to understand variation in helping behaviour in cooperative breeders.

The potential benefits to helpers deriving from breeders depend on the characteristics of the breeders they are helping (e.g. sex, relatedness). For instance, related breeders can be associated with future indirect benefits for helpers [20,23,24] and benefits of prolonged parental nepotism [25–27]. Unrelated opposite-sex breeders on the other hand may offer current or future mating benefits to helpers [10,28,29], whereas unrelated same-sex breeders offer no benefits and instead may compete for reproduction [30]. The characteristics of breeders in relation to helpers can vary substantially among and within cooperative breeders [7]. Therefore, more comprehensive analyses of helping behaviour are required that include benefits from positive effects on breeders, taking into account the type of breeders that helpers are assisting. This will yield important insights into the importance of benefits deriving from enhanced fitness of breeders for the evolutionary maintenance of helping behaviour.

Here, we test whether variation in the value of breeders to helpers explains the variation in help with offspring provisioning in a facultatively cooperative breeding bird, the purple-crowned fairy-wren, *Malurus coronatus*. Groups are formed by a dominant pair, usually with additional subordinates, which may or may not help provision the dominant pair's offspring [10,16]. Social group composition varies: subordinates can be related or unrelated to the brood as well as to breeders, owing to breeder turnover and subordinate dispersal [10,30,31]. Breeders decrease their provisioning rates when receiving help (although total provisioning rates nonetheless increase), resulting in increased survival and reproductive output [16]. Subordinates benefit from sharing a group with breeders that are kin and/or potential mates (unrelated, opposite-sex): these offer benefits from mutualistic social bonds, as well as the potential for breeding position inheritance and kin-selected benefits [10,30,31]. Unrelated same-sex breeders on the other hand represent reproductive competitors that offer no benefits [30].

Previous analyses taking into account helping benefits deriving from the brood only, showed that offspring provisioning by subordinate purple-crowned fairy-wrens is driven by indirect benefits (from helping raise relatives) and active group augmentation benefits (through breeding position inheritance), acting in interaction [10]. Here we repeat, expand and refine this study, first, because changed ecological conditions (notably increased population density and number of helpers per group) have altered the probabilities and potential importance of breeding position inheritance for subordinates [10,31]; second, because our understanding of purple-crowned fairy-wren social behaviour has improved, particularly in relation to social group composition [30,31]; and third, to contribute to the replication effort in ecological science [32]. Our aim is to reassess the drivers of subordinate help with offspring provisioning, and to differentiate between various benefits deriving from the brood and from supporting valuable breeders, which has not previously been tested. Nuanced analyses in other contexts have revealed how social context may drive the behaviour of subordinates in this species. Subordinates form close social bonds and engage in frequent affiliation with group members that are kin and potential mates, while interactions with reproductive competitors are antagonistic [30]. Similarly, when a group member is at risk of attack by a predator, subordinates predominantly engage in dangerous predator defence if this group member is kin or a potential mate [31]. Therefore, we predict that help with offspring provisioning may also serve to support valuable breeders, i.e. kin and/or potential mates. Here, we test for such an effect of social context on helper contribution to offspring provisioning, while controlling for any potential fitness benefits deriving from the brood that have been tested previously (indirect benefits, benefits from enhanced group size and/or gaining social prestige; see table 1 for full overview of hypotheses and predictions). Using this integrative approach, we highlight that neglecting the benefits that breeders provide to helpers can lead to misinterpretation of results, since prevailing hypotheses based on benefits deriving from the brood alone cannot fully explain the extensive variation in helping effort.

**Table 1.** Support for our hypothesis that helping serves to support valuable breeders. (Presented are all proposed hypotheses for the evolutionary maintenance of cooperative breeding that could apply to purple-crowned fairy-wrens^a^ along with an explanation (principle), the predicted investment in offspring provisioning by subordinate purple-crowned fairy-wrens according to each hypothesis, and whether they were supported in our study. Hypothesis 1, which explicitly considers social context, is newly proposed and tested here and supported by our findings: helpers preferentially provision offspring of breeders that represent a potential mate and/or (a) relative(s). Hypotheses 2–5 are directly related to benefits associated with the production of additional recruits (hypotheses 2–4), or gained through the act of helping itself in the presence of a potential mate (hypothesis 5).)

hypothesis^a^	principle	predictions in *M. coronatus*	supported?
1. support of valuable breeders	enhancing fitness of valuable breeders yields indirect and/or direct benefits to subordinates	provisioning rates are higher when one of the breeders is a potential future mate (unrelated, opposite-sex)^b^	yes, partly (only if the same-sex breeder is a relative; figure 1; table 2*a*)
		provisioning rates are higher when one or both breeders are relatives (independent of breeder sex)^b^	yes (figure 1; table 2*a*)
2. kin selection [23]	increasing the production of related offspring yields indirect fitness benefits	subordinates do not feed unrelated nestlings	yes (figure 2; table 2*b*)
		provisioning rates are higher with higher relatedness to the brood	no (figure 2; table 2*b*)
3. passive group augmentation [13]	increasing group size enhances subordinates' chances of survival owing to the mere presence of additional individuals	provisioning rates decrease with group size^c^	no (table 2)
4. active group augmentation [13]	increasing group size enhances future help received from recruits when subordinates obtain the breeding position	provisioning rates increase with probability of breeding position inheritance^d^	no (table 2)
5. social prestige [33]	helping signals quality to potential future mates	provisioning rates are higher when a subordinate potential mate is present^e^	no (table 2)

^a^Several hypotheses can be ruled out as they are not applicable in this species: helping is unlikely to be driven by opportunities for direct access to reproduction (parentage acquisition hypothesis; [1,28]), or to serve as payment of rent (pay-to-stay hypothesis; [2,14]), as helpers rarely gain paternity or lay eggs in this species [34], and non-cooperative individuals are tolerated in the territory [10,16,35]. See also Teunissen *et al.* [31] for similar rationale.

^b^Related breeders lead to kin-selected benefits and benefits of parental nepotism; potential mates offer potential mating benefits through breeding position inheritance; both relatives and potential mates offer benefits from mutualistic social bonds [30].

^c^Benefits of increasing group size are expected to be greater in small compared to large groups, owing to diminishing returns of additional group members [6].

^d^Subordinates can only benefit from recruits helping with breeding in the future if they can inherit a breeding position in the territory, thus helping effort should be related to chances of breeding position inheritance [6,13].

^e^Since queues for breeding position inheritance are stable [10,31], helping is unlikely to serve as a signal of quality to breeders, but may serve to advertise quality to subordinate potential mates with which to establish a new territory (as 7% of subordinates do; [36]).

## 2. Methods

### 2.1. Study site and species

Purple-crowned fairy-wrens are endemic to the wet–dry tropics of northern Australia, where they inhabit riparian vegetation. Groups and territories are stable year-round and consist of a monogamous dominant breeding pair (which engage in duetting behaviour, providing a reliable cue to assign social status [37]) and often one or more subordinates [10,16]. Extra-pair paternity is rare (4.4% of offspring), and subordinates rarely gain direct access to reproduction (1.8% of offspring [10,34]). Breeding vacancies are rare, and the cost of inbreeding is high [38]. Subordinates can inherit a breeding position in the group when the same-sex breeder disappears (20% of breeding positions obtained this way), or in rarer cases, pair with an unrelated opposite-sex subordinate in the group and bud off to form a new territory (7% of breeding positions [36]). Subordinates of both sexes can help the breeding pair to raise offspring, and vary in offspring provisioning rates [10]. Most breeding takes place during the monsoonal wet season (December–March [39]).

We studied a population of approximately 300 uniquely colour-banded individuals, monitored since 2005 at Australian Wildlife Conservancy's Mornington Wildlife Sanctuary in northwest Australia (17°31′ S, 126°6′ E). For the current study, 50 fairy-wren groups were followed closely during three main breeding seasons (during the wet season) from 2016 to 2018, recording individuals' social status (dominant breeder or subordinate) and breeding activity. Nests were monitored for egg laying, hatching, and fledging. *Malurus coronatus* generally lay 3 or 4 eggs (range 1–5, mean ± s.e. = 3 ± 0.03), and nestlings fledge approximately 13 days after hatching [10,40].

### 2.2. Nestling provisioning observations

We quantified individual nestling provisioning rate for 71 subordinates in February–April 2016, March–April 2017 and December 2017–April 2018 (year ‘2016’, ‘2017’ and ‘2018’, respectively). We conducted 77 nestling provisioning watches at 45 nests on 32 territories containing subordinate group members, resulting in a dataset of 145 individual observation hours. Two 1 h provisioning watches were conducted at each nest where possible (owing to nest failure, some nests were only watched once; mean watches per nest ± s.e. = 1.7 ± 0.1), between 3 and 8 days after hatching (mean days post-hatching ± s.e. = 5.6 ± 0.2). Previous research on this study population indicated that two 1 h observations are sufficient to estimate helping effort [35]. Provisioning watches were conducted during the morning (starting between 05.25 and 10.25) during calm, dry weather, from a camouflaged hide approximately 10 m from the nest. The identity of each bird bringing food to the nestlings was recorded. Group members could be unambiguously identified by their colour bands in 96% (937 of 974) of feeding visits. Only individuals older than 94 days, the youngest age of a bird that was seen provisioning, were considered as focal subordinates. The youngest age of a subordinate that was unrelated to one or more of the breeders in our dataset was 82 days.

### 2.3. Statistical analyses

Analyses were performed in R 3.4.4 [41]. Subordinates did not feed nestlings when they were unrelated to the brood, creating complete separation of the data (figure 2). Therefore, we analysed subordinate provisioning rates at the nest with Bayesian generalized linear mixed models with a negative binomial distribution using the ‘rstanarm’ package [42]. We fitted models including the number of feeds at the nest by the focal subordinate during a 1 h nest watch as response variable (*n* = 145 observations on 71 subordinates). We included the following covariates known to explain variation in provisioning rates by subordinates: sex (male, female), age (first-year, older), brood size, brood age (days) and time of day (h). Year (2016, 2017, 2018) was also included to account for variation across years. Bird identity (ID) and nest ID were included as random effects to account for replication across individuals and nests. Priors were set to a normal distribution with mean = 0 and variance = 10 and variance = 100 for the intercept prior. Three chains were run of 15 000 iterations each, with a thinning interval of 20 and a warm-up period of 5000. Visual inspection of the trace, density, autocorrelation and posterior predictive plots using the ‘rstan’ package [43] confirmed convergence of the model and showed no sign of autocorrelation. We present posterior means and 95% credible intervals (CIs) for all effects.

We quantified potential benefits of helping as proposed by each of the hypotheses and their predictions outlined in table 1 as follows:

(i) benefits from supporting valuable breeders depend on the type of breeders subordinates could assist, which is determined by sex and relatedness [30]. The same-sex breeders were classified as either a reproductive competitor (if unrelated) or a relative. Opposite-sex breeders were classified as a potential mate (if unrelated) or a relative;
(ii) indirect benefits from kin selection were quantified as focal subordinates' relatedness to the brood, classified as *r* = 0 (unrelated to both breeders), *r* = 0.25 (related to one breeder only) or *r* = 0.50 (related to both breeders);
(iii) passive group augmentation benefits were quantified as group size at the start of breeding (mean group size ± s.e. = 3.8 ± 0.2, range = 3–7);
(iv) active group augmentation benefits were quantified as subordinates’ likelihood to inherit the breeding position on the territory should a vacancy open up (i.e. when the same-sex breeder disappears from the territory). This depends on focal subordinates' sex (males have slightly higher chances to inherit the breeding position), relatedness to the opposite-sex breeder (subordinates are more likely to inherit the breeding position when unrelated to the opposite-sex breeder), and whether an older same-sex subordinate group member is present (which greatly reduces the chances of breeding position inheritance) [10,31]. Inheritance probabilities were calculated using inheritance data from 2012 to 2018 for the entire population and range from 0% to 93%; see [31] for a detailed description of how breeding position inheritance probabilities were calculated; and
(v) benefits from social prestige were determined by the presence of an unrelated opposite-sex subordinate in the group, classified as present or absent.

Since extra-pair paternity is 4.4% [34], social relatedness reflects genetic relatedness in this species. Relatedness of subordinates to breeders and to subordinate group members was, therefore, determined using social pedigree data. Only first-order relatives (full sibling, parent–offspring) were classified as ‘related’ group members. Our dataset contained six cases where a focal subordinate was a second-order relative (e.g. half-sibling, cousin) of a group member; these were classified as ‘unrelated’ for analyses.

We tested whether subordinates help feed nestlings to support specific, valuable, breeders (table 1, hypothesis 1), while simultaneously including any potential fitness benefits deriving from the production of additional recruits or directly from helping itself that may additionally or alternatively drive helping behaviour. However, since subordinates' relatedness to the brood is determined by the type of breeders they assist (i.e. whether they are related to breeders), we could not test for the support of valuable breeders hypothesis and the kin selection hypothesis in the same model. Instead, we ran two separate models (A and B) to statistically disentangle the effect of relatedness of subordinates to breeders (hypothesis 1) from the relatedness to the brood (hypothesis 2). Therefore, model A included as independent variables: the value of the opposite-sex breeder (potential mate versus relative) and of the same-sex breeder (reproductive competitor versus relative) to subordinates, and their interaction (hypothesis 1); group size (hypothesis 3); probability of breeding position inheritance (hypothesis 4), and the presence of a subordinate potential mate (hypothesis 5). Model B included relatedness to the brood (hypothesis 2), group size (hypothesis 3); probability of breeding position inheritance (hypothesis 4), and the presence of a subordinate potential mate (hypothesis 5).

Although relatedness to the brood (kin selection) and probability of inheritance (active group augmentation) have previously been found to affect provisioning rates by helper purple-crowned fairy-wrens in interaction [10], we were unable to include such an interaction effect in our models because subordinates never fed unrelated broods. Instead, we re-ran the model B analysis for data subsets where focal subordinate relatedness to the brood was either 0.25 or 0.5, to test for an effect of probability of inheritance at different levels of relatedness to the brood, and thus verify that our findings do not reflect an undetected interaction. Both models confirmed a lack of effect of breeding position inheritance probability on nestling provisioning rates at these levels of relatedness (similar to our other models; table 2), suggesting there is no hidden interacting effect of relatedness to the brood and inheritance probability.

**Table 2.** Investment in offspring provisioning by subordinates is most clearly explained by the value of the breeders they are helping. ((*a*) Results of statistical model A including benefits associated with support of valuable breeders, passive and active group augmentation, and social prestige. Widely applicable information criterion (WAIC) = 377.3. (*b*) Results of statistical model B including benefits from raising related young (kin selection), passive and active group augmentation and social prestige. WAIC = 376.5. Both models explain variation in helping effort equally well (ΔWAIC = 0.8). They indicate that variation in helping effort is explained by subordinates’ relatedness to the brood, but not as predicted by kin selection theory alone. By explicitly considering the type of breeders that subordinates assist, the finding that subordinates feed nestlings most often when these are second-order relatives can be explained. Significant terms are highlighted in italics.)

	posterior mean (95% CI)
(*a*)	
intercept	−5.0 (−13.6, 2.4)
*same-sex breeder* (*relative*)^a^	*7.3* *(2.8, 14.1)*
*opposite-sex breeder* (*relative*)^b^	*6.1* *(1.3, 13.2)*
*same-sex* (*relative*)^a^ *x opposite-sex breeder* (*relative*)^b^	*−8.7* *(−15.7, −3.7)*
probability of breeding position inheritance	−0.7 (−2.7, 1.2)
subordinate potential mate (present)^c^	0.4 (−1.0, 1.8)
group size	−0.3 (−1.0, 0.4)
sex (male)^d^	0.2 (−1.0, 1.4)
subordinate age (>1)^e^	−0.6 (−2.2, 0.8)
brood size	0.2 (−0.5, 0.9)
brood age	*0.3* *(0.1, 0.5)*
year (2017)^f^	−2.3 (−4.7, 0.1)
year (2018)^f^	−1.2 (−3.5, 1.1)
time of day	−0.1 (−0.5, 0.2)
(*b*)	
intercept	−6.7 (−18.1, 1.8)
*relatedness to brood* (*r = 0.25*)^g^	*8.7* *(3.0, 18.6)*
*relatedness to brood* (*r = 0.5*)^g^	*6.3* *(0.4, 16.1)*
probability of breeding position inheritance	−0.7 (−2.6, 1.1)
subordinate potential mate (present)^c^	0.3 (−1.0, 1.6)
group size	−0.2 (−0.9, 0.4)
sex (male)^d^	0.1 (−1.0, 1.3)
subordinate age (>1)^e^	−0.5 (−2.2, 0.9)
brood size	0.2 (−0.5, 0.8)
*brood age*	*0.3* *(0.1, 0.5)*
*year* (*2017*)^f^	−*2.4* *(−4.9, −0.2)*
year (2018)^f^	−1.3 (−3.7, 0.9)
time of day	−0.1 (−0.5, 0.3)

^a^Reference value is reproductive competitor.

^b^Reference value is potential mate.

^c^Reference value is ‘absent’.

^d^Reference value is female.

^e^Reference value is less than 1 year old.

^f^Reference value is 2016.

^g^Reference value is *r* = 0.

## 3. Results

### 3.1. Benefits driving helping behaviour

**Figure 1.**
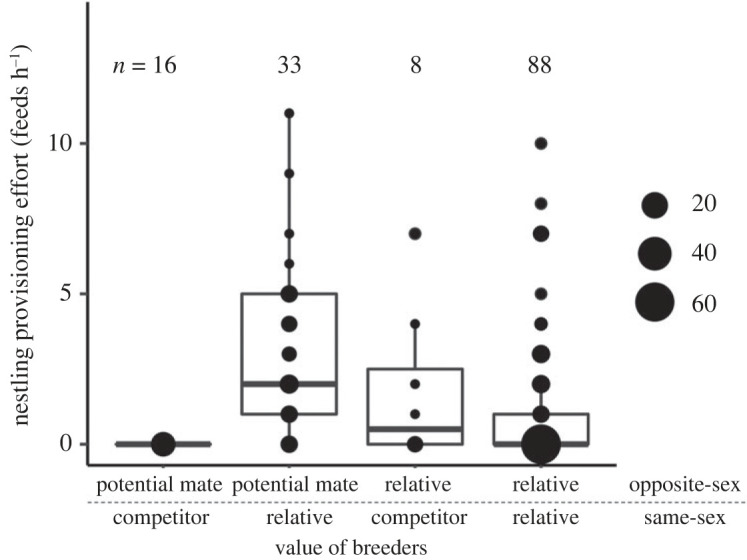
Subordinates provision nestlings most when they are assisting valuable breeders associated with multiple benefits: a potential mate and a relative. An interacting effect of the value of the same-sex and the opposite-sex breeder to subordinates indicates that subordinates help less when assisting a relative and a competitor or two relatives, with no help provided when the breeding pair consists of a potential mate and a competitor. Dots depict the raw values; area of dots indicates the number of data points at each value. Bold horizontal lines reflect the median provisioning rates and whiskers indicate 95% CIs. Numbers above boxplots indicate sample sizes.

The type (i.e. value) of breeders that subordinates assisted (hypothesis 1) explained variation in helping effort (table 2*a*). Relatedness to breeders positively affected helping effort: provisioning rates were higher when the same-sex breeder or the opposite-sex breeder represented a relative (main effect of both; table 2*b*). Moreover, the value represented by the same-sex and the opposite-sex breeder had interacting effects on subordinate provisioning rates (table 2*a*). When a breeding pair represented a reproductive competitor (of no or negative value) and a valuable potential mate (i.e. both were unrelated), subordinates did not feed nestlings at all. When both breeders were relatives, they fed at similar rates compared to when they were assisting a relative and a competitor. However, subordinates fed nestlings more than twice as often when they were assisting a relative *and* a potential mate (figure 1).

**Figure 2.**
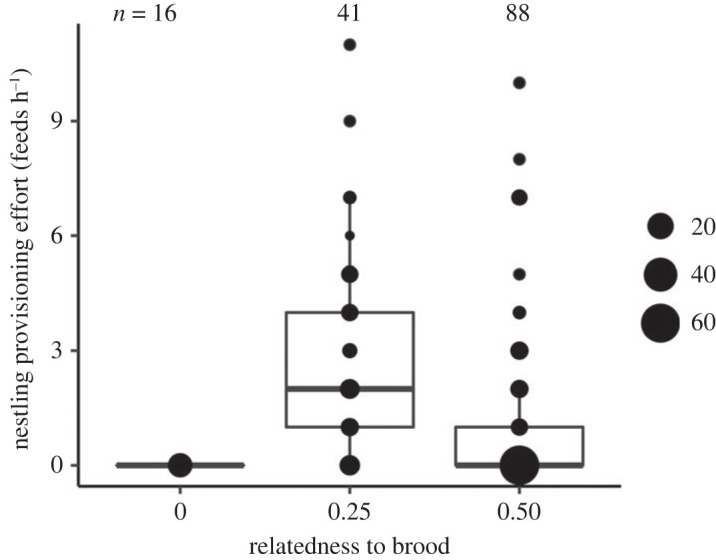
Individual nestling provisioning rates by subordinate purple-crowned fairy-wrens vary with their relatedness to the brood but not as predicted by kin selection theory, suggesting that other benefits rather than offspring relatedness drive this pattern. Subordinates that were unrelated to the brood (*r* = 0) never fed nestlings, and subordinates that were raising half-siblings (*r* = 0.25) fed most often. Dots depict the raw values; area of dots indicates the number of data points at each value. Bold horizontal lines reflect the median provisioning rates and whiskers indicate 95% CIs. Numbers above boxplots indicate sample sizes.

Variation in helping effort was also explained by subordinates’ relatedness to the brood, but not as predicted by kin selection theory (hypothesis 2; table 2*b*). While subordinates never fed unrelated nestlings, surprisingly, subordinates fed nestlings more frequently when they were raising second-order relatives (*r* = 0.25) compared to first-order relatives (*r* = 0.50) (*post hoc* test: posterior mean (CI) of *r* = 0.25 relative to *r* = 0.50 = 2.3 (0.3, 4.2); table 2*b*; figure 2; note that this was not a ‘hidden’ age effect since we corrected for age in the model). Thereby our results support only one of the two predictions for kin selection theory (prediction 2.1 in table 1). Instead, the finding that subordinates fed second-order relatives more often can only be explained by the characteristics of breeders (see above).

Helping effort was not explained by passive group augmentation benefits, as helping effort did not vary with group size (hypothesis 3; table 2), nor was it affected by active group augmentation benefits (no effect of breeding position inheritance probability; hypothesis 4; table 2). We also found no effect of the potential to gain social prestige by advertising to a subordinate potential mate (hypothesis 5; table 2).

## 4. Discussion

We provide a comprehensive test of the drivers of helping behaviour by subordinates in a cooperative breeder, while accounting for all potential indirect and direct benefits that can accrue through associations with breeders, enhanced production of offspring, or the act of helping itself. We show that offspring provisioning by subordinates is not driven by the direct fitness benefits that are commonly proposed (passive or active group augmentation, social prestige), nor is it completely explained by indirect benefits associated with enhanced production of related offspring (kin selection). Instead, our findings can only be explained when considering the type of breeders that subordinates assist (i.e. determined by sex and relatedness). Our findings indicate that subordinates assist most with offspring provisioning when they are helping breeders that provide indirect kin-selected, nepotistic and future potential mating benefits. This supports our hypothesis that helping behaviour may be driven by benefits associated with supporting specific, valuable, breeders.

### 4.1. Producing additional recruits or gaining social prestige

All commonly proposed hypotheses that explain helping behaviour based on direct fitness benefits derived from the brood being raised did not clearly explain variation in offspring provisioning. First, helpers rarely gain parentage [34], ruling out direct access to reproduction as a driver of helping (parentage acquisition [1,44]). Second, dominant breeders tolerate subordinates that do not provide help [10,35], ruling out the need to provide help to be able to stay in the group (pay-to-stay [2,14]). Third, provisioning rates did not vary with group size, indicating that investment in offspring provisioning is not driven by benefits resulting from larger group size (passive group augmentation [6,13]). Fourth, we found no effect of subordinates' probability of inheriting a breeding position on the territory, suggesting that helping raise offspring does not serve to enhance the production of recruits that might in the future act as helpers themselves should the subordinate obtain a breeding position in the group (active group augmentation [6,13]). Lastly, provisioning rates did not increase when a (subordinate) potential mate was present, suggesting that helping does not serve as an advertisement signal either (social prestige [3,33,45]). For all the above hypotheses, it should be noted that helpers could adjust their effort not only through feeding frequency, but through for example type of prey items delivered instead, which we did not test for.

Our study found clear support for kin selection explaining helping behaviour, since no subordinate ever helped to raise unrelated broods [23,46]. Kin selection is often shown to explain helping behaviour within and between species [3–5,47]. However, it explains only a small proportion of variation in helping effort among cooperative breeders and is often not the only driver of helping [4,9,10,12]. Similarly in our study, provisioning rates did not increase when subordinates shared a higher degree of relatedness to the brood, and the indirect fitness benefits of increased production of offspring were thus greater (in contrast to e.g. [4,5,47–49]). Instead, subordinates fed half-siblings more frequently than full siblings, and therefore, kin selection theory alone cannot explain variation in helper provisioning effort in this species.

### 4.2. Supporting valuable breeders

The fact that nestling provisioning by subordinates also benefits breeders, through improved survival and/or reproduction, is widespread in cooperative breeders [16,17,20,21,50]. Yet, helpers’ stake in breeders is rarely considered as a motivation for subordinates to help. By taking into account the sort of breeders that helpers were assisting, we showed that helpers assisting a same-sex relative and an opposite-sex potential mate worked the hardest (figure 1). Potential mates are associated with future reproductive benefits ([10,31]; see also [28,51]), and mutually beneficial social bonds [30]. Since breeding vacancies are rare and the cost of inbreeding is high [38], such unrelated opposite-sex breeders are highly valuable for when opportunities to achieve dominance in the territory arise. Related breeders in turn offer indirect kin-selected benefits, potentially benefits of parental nepotism, and also mutualistic social bonds [30]. Our findings thus seem to suggest that offspring provisioning by helpers is driven by both indirect kin-selected benefits from enhancing fitness of related breeders, and by direct benefits associated with enhanced fitness of potential mates, in interaction.

Alternatively, helping may not necessarily be driven by effects on breeder fitness directly, but instead subordinates may be choosing to help breeders they share close social bonds with. Social bonds themselves are beneficial and these breeders may in turn provide other reciprocal benefits [30,52–54]. Additionally, it is possible that maintaining a strong social bond with a breeder who represents a potential future mate may increase subordinates' chances of mating with them in the future. Whichever the mechanism, our findings indicate that subordinates are specific in which type of breeders they help. This aligns with recent findings from our study population showing that investment in defence by subordinates to a dangerous predator is similarly driven by benefits associated with survival of adult group members that are relatives or potential mates [31]. These findings hence highlight the importance of social context in driving cooperative behaviours.

The fact that subordinate fairy-wrens in our study never fed nestlings when they were unrelated to both breeders, despite one of them being a potential mate, could suggest that kin-selected benefits may be a prerequisite for helping with offspring provisioning. However, an alternative explanation for this finding is that it is a result of social conflict: a breeder may simply not tolerate an unrelated same-sex subordinate near the nest if it is also unrelated to the other breeder, i.e. represents a potential mate and therefore poses a threat to the pair bond. Indeed, while such conflict rarely manifests in aggression in purple-crowned fairy-wrens, aggression is usually directed from breeders to unrelated same-sex subordinates [30]. Hence, our results suggest that there is probably substantial conflict between breeders over competitive subordinates in the group (as may also be the case in other cooperative breeders, e.g. [55]), to the extent that they may be prevented from helping at the nest altogether, further highlighting the importance of considering social context in order to understand cooperative behaviours.

### 4.3. Changes in drivers of helping over time

Our findings are only partly in line with previous research on this species. Although these previous analyses of similar data did not explicitly test for valuable breeders, they showed that offspring provisioning by subordinates was driven by indirect kin-selected benefits (hypothesis 2 in table 1) as well as active group augmentation benefits (breeding position inheritance; hypothesis 4) acting in interaction [10]. While we found some effect of indirect fitness benefits, we did not detect higher provisioning rates with increased chances of breeding position inheritance, but instead, only when one of the breeders was a potential mate. This suggests that maintaining social bonds with (and perhaps enhancing fitness of) potential mates, rather than future help provided by additional recruits, is currently driving helping behaviour in the population. Possibly, this reflects how ecological conditions have changed at our field site over time. Fairy-wren density has increased by 70%; from a mean ± s.e. of 10 ± 0.6 individuals km^−1^ in 2005–2009, the study period used by Kingma *et al*. [10], to 17 ± 1.3 individuals km^−1^ in 2016–2018 during this study. Concurrently, the number of subordinates in our study population has increased by over 85% (from a mean ± s.e. of 60 ± 5.6 in 2005–2009 to 113 ± 20 in 2016–2018), and the number of territories by 54% (from 48 ± 3.5 to 74 ± 0.6), to seemingly stable levels indicating saturation of the population (N. Teunissen, A Peters 2023, unpublished data). We propose here that a shortage of vacant breeding positions might result in subordinates staying on the resident territory longer. This may explain why benefits associated with breeders now appear to be more important in driving offspring provisioning by helpers than benefits associated with the production of additional offspring. These different findings to Kingma *et al.* [10] highlight the value of replicating studies [32], as different results may be obtained on the same species or even study population when ecological conditions change. The changes in helping behaviour and social dynamics over time, and their effect on fitness of individuals of different status in the population will be subject to further study.

## 5. Conclusion

Commonly proposed adaptive hypotheses for the evolution of helping behaviour do not fully explain offspring provisioning effort by subordinate purple-crowned fairy-wrens. By taking into account the type of breeders that subordinates assist, we show that indirect and direct fitness benefits associated with valuable breeders drive helping behaviour. Load-lightening appears common among cooperative breeders [18,20,22,56], and social group composition varies considerably between and within species [7]. Therefore, it seems imperative to incorporate benefits that breeders might offer to helpers at present or in future in studies on cooperative behaviour, to achieve a comprehensive understanding of the complexity of the evolutionary maintenance of helping behaviour in cooperative breeders.

## Data Availability

The data and code used for statistical analyses are provided in the electronic supplementary material [57].
